# Zika virus preferentially replicates in the female reproductive tract after vaginal inoculation of rhesus macaques

**DOI:** 10.1371/journal.ppat.1006537

**Published:** 2017-07-26

**Authors:** Timothy Carroll, Ming Lo, Marion Lanteri, Joseph Dutra, Katie Zarbock, Paola Silveira, Tracy Rourke, Zhong-min Ma, Linda Fritts, Shelby O’Connor, Michael Busch, Christopher J. Miller

**Affiliations:** 1 Center for Comparative Medicine University of California, Davis, Davis, California, United States of America; 2 California National Primate Research Center, University of California, Davis, Davis, California, United States of America; 3 Blood Systems Research Institute, San Francisco, California, United States of America; 4 Wisconsin National Primate Research Center, University of Wisconsin, Madison, Wisconsin, United States of America; 5 Federal University of Rio de Janeiro, Rio de Janeiro, Brazil; NIH, UNITED STATES

## Abstract

Zika virus (ZIKV) is a mosquito-transmitted virus that can cause severe defects in an infected fetus. ZIKV is also transmitted by sexual contact, although the relative importance of sexual transmission is unclear. To better understand the role of sexual transmission in ZIKV pathogenesis, a nonhuman primate (NHP) model of vaginal transmission was developed. ZIKV was readily transmitted to mature cycling female rhesus macaque (RM) by vaginal inoculation with 10^4^–10^6^ plaque-forming units (PFU). However, there was variability in susceptibility between the individual RM with 1–>8 vaginal inoculations required to establish infection. After treatment with Depoprovera, a widely used contraceptive progestin, two RM that initially resisted 8 vaginal ZIKV inoculations became infected after one ZIKV inoculation. Thus, Depoprovera seemed to enhance susceptibility to vaginal ZIKV transmission. Unexpectedly, the kinetics of virus replication and dissemination after intravaginal ZIKV inoculation were markedly different from RM infected with ZIKV by subcutaneous (SQ) virus inoculation. Several groups have reported that after SQ ZIKV inoculation vRNA is rapidly detected in blood plasma with vRNA less common in urine and saliva and only rarely detected in female reproductive tract (FRT) secretions. In contrast, in vaginally inoculated RM, plasma vRNA is delayed for several days and ZIKV replication in, and vRNA shedding from, the FRT was found in all 6 animals. Further, after intravaginal transmission ZIKV RNA shedding from FRT secretions was detected before or simultaneously with plasma vRNA, and persisted for at least as long. Thus, ZIKV replication in the FRT was independent of, and often preceded virus replication in the tissues contributing to plasma vRNA. These results support the conclusion that ZIKV preferentially replicates in the FRT after vaginal transmission, but not after SQ transmission, and raise the possibility that there is enhanced fetal infection and pathology after vaginal ZIKV transmission compared to a mosquito transmitted ZIKV.

## Author summary

Zika virus was introduced to Brazil in 2015 and it rapidly spread to all of tropical America. Although Zika virus infection is usually mild in adults, it can cause severe birth defects in the developing fetus that makes it critical to prevent ZIKV infection in women who are pregnant or who could become pregnant. Although Zika virus is transmitted primarily by mosquito bite, it can also be transmitted by sex. To understand the role of sexual transmission in Zika virus disease, we inoculated rhesus monkeys intravaginally with the virus and monitored virus in blood and reproductive tract secretions. ZIKV was detected in the female reproductive tract before it was detected in plasma and replication levels in the female reproductive tract did not reflect ZIKV levels in other parts of the body. Thus ZIKV prefers the reproductive tract after vaginal transmission suggesting that fetal disease could be more common or severe after vaginal ZIKV transmission compared to a mosquito transmitted ZIKV infection.

## Introduction

Zika virus (ZIKV) was first isolated in the Zika forest of Uganda in 1947 (21, 22, 30) and the first descriptions of human disease were reported a few years later (2, 53). ZIKV has a positive-sense RNA genome and belongs to the genus Flavivirus, which also includes dengue virus (DENV), Yellow Fever virus, Japanese encephalitis virus, and West Nile virus (WNV) (30). In approximately 20% of infected humans, ZIKV causes a febrile illness that can include rash, arthralgia and conjunctivitis. In addition, ZIKV has been associated with the development of microcephaly and lissencephaly and ocular lesions in infants born to women who acquired the infection during early pregnancy. In adults, ZIKV infection has also been associated with Guillan-Barré syndrome and other neurological complications including hearing loss and tinnitus. Although ZIKV is a mosquito-transmitted virus, sexual transmission of ZIKV in humans has been documented in several settings [1–13]. After returning to the U.S. from Africa, a man infected his partner [2] and male-to-female [14], male-to-male [5] and female-to-male [10] sexual transmission of ZIKV have been reported in travelers returning to the U.S. from ZIKV positive regions in the Americas. ZIKV was isolated from semen during the ZIKV outbreak in French Polynesia in 2013 [4] and infectious virus has been isolated from semen up to 24 days after the onset of symptoms [9]. Further, ZIKV RNA has been detected in semen up to 6 months after onset of symptoms [15,16] and in the semen of a vasectomized man up to 96 days after onset of symptoms [6]; however, the infectivity and transmission potential of persistent ZIKV RNA in semen is not known. Of significant concern, a case of male-to-female sexual transmission of ZIKV from an asymptomatic male traveler to a woman with no travel history has been reported [8]. This case suggests that transmission via semen is possible even if a man has minimal or no symptoms.

In 2007, an Asian lineage ZIKV outbreak from mosquito transmission was reported in Yap Island with 185 clinical cases and an estimated 5000 infections (75% of the population) in just 3 months [17,18]. Six years later (in 2013), another ZIKV outbreak involving 28,000 infected people was reported approximately 5000 miles away in French Polynesia (FPY) [19]. The ZIKV strain in the FPY outbreak had 99.9% nucleotide and amino acid identities with the Asian ZIKV strain in the Yap Island outbreak [17,19,20], suggesting that the virus in French Polynesia outbreak was imported from Yap Island. Given the distance between the two locations it is unlikely that mosquitoes introduced ZIKV into FPY; it is more likely that an infected person imported ZIKV to FPY. ZIKV subsequently spread from FPY to other Pacific Islands, and by 2014 imported cases and cases of autochthonous transmission were reported in New Caledonia, Easter Island and the Cook Islands [21,22]. The nucleotide sequence of the ZIKV strain in all these outbreaks was 99.9% identical to the ZIKV strain in the Yap Island and FPY outbreaks. In March 2015, the first cases of autochthonous transmitted ZIKV were reported in Bahia, Brazil with a ZIKV strain that was 99.9% identical (nucleotide and aa sequences) to the ZIKV strain in the Yap Island and FPY outbreaks [23,24]. Based on this chain of events and the similarity of the ZIKV strains involved, it is generally accepted that ZIKV moved from the Pacific Islands to South America. ZIKV mosquito vectors are endemic in the Pacific Islands and Brazil [25,26] and ZIKV is readily transmitted between humans by sexual activity [1–13]. Thus, it is likely that one or more infected individuals imported ZIKV over considerble distances to these widely separated islands and countries and then served as reservoir hosts for mosquito transmission, or transmitted ZIKV by sex, to naïve persons.

The World Health Organization declared the ZIKV pandemic a public health emergency on February 1, 2016, and in November 2016, WHO declared Zika virus endemic in the Americas. As of May 2017, more than 5,109 cases of ZIKV infection have been reported in the United States, excluding those in Puerto Rico, Virgin Islands and Guam. Most infections are in travelers returning from affected areas, but 266 ZIKV infections were acquired in the continental US. Of these US acquired infections, 221 infections (83%) were transmitted through mosquito bites in Florida and Texas, while 45 infections (17%) were sexually transmitted [27]. It is now estimated that 1.6 million people are, or have been, infected with ZIKV in the Americas. Despite these observations, the frequency and efficiency of sexual ZIKV transmission is unclear. To better understand the biology of ZIKV sexual transmission, we developed a RM model of vaginal ZIKV transmission.

## Materials and methods

### Ethics statement

The captive-bred mature (> 5year old) parous, cycling female rhesus macaques (Macaca mulatta) used in this study were from the California National Primate Research Center. All animals were negative for antibodies to WNV, HIV-2, SIV, type-D retrovirus, and simian T cell lymphotropic virus type 1 at the time the study was initiated. The animals were housed in accordance with the recommendations of the Association for Assessment and Accreditation of Laboratory Animal Care International Standards and with the recommendations in the Guide for the Care and Use of Laboratory Animals of the National Institutes of Health. The Institutional Animal Use and Care Committee of the University of California, Davis, approved these experiments (Protocol # 19471). When immobilization was necessary, the animals were injected intramuscularly with 10 mg/kg of ketamine HCl (Parke-Davis, Morris Plains N.J.). All efforts were made to minimize suffering. Details of animal welfare and steps taken to ameliorate suffering were in accordance with the recommendations of the Weatherall report, "The use of non-human primates in research". Animals were housed in an air-conditioned facility with an ambient temperature of 21–25°C, a relative humidity of 40%-60% and a 12 h light/dark cycle. Animals were individually housed in suspended stainless steel wire-bottomed cages and provided with a commercial primate diet. Fresh fruit was provided once daily and water was freely available at all times. A variety of environmental enrichment strategies were employed including housing of animals in pairs, providing toys to manipulate and playing entertainment videos in the animal rooms. In addition, the animals were observed twice daily and any signs of disease or discomfort were reported to the veterinary staff for evaluation. The menstrual cycles were assessed on the basis of menstrual bleeding, with the first day of menses designated day 0 of the cycle. For sample collection, animals were anesthetized with 10 mg/kg ketamine HCl (Park-Davis, Morris Plains, NJ, USA) or 0.7mg/kg tiletamine HCl and zolazepan (Telazol, Fort Dodge Animal Health, Fort Dodge, IA) injected intramuscularly. The animals were sacrificed by intravenous administration of barbiturates.

Plasma from a ZIKV infected blood donor was used to produce the ZIKV stock for these studies. The donated blood was collected at the Hematology and Transfusion Center, Hospital of Clinics, Universidade Estadual de Campinas-UNICAMP, Campinas, SP, Brazil, and after it was found to positive for ZIKV by RT-PCR, the fresh frozen plasma was released for research. However, no donor personal identification information accompanied the sample and thus, the donor is anonymous [28] and IRB approval was not needed to isolate virus from the sample.

### Vaginal ZIKV inoculation

**Fig 1:**
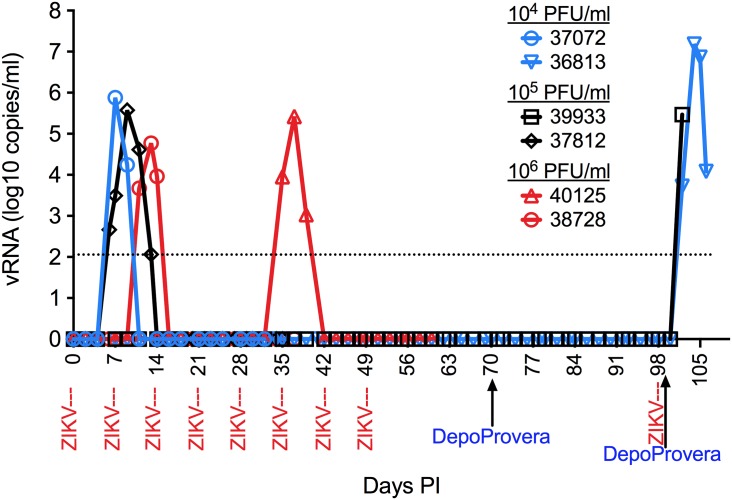
Overview of vaginal ZIKV transmission studies in RM. Plasma ZIKV RNA levels. Each weekly ZIKV inoculation is indicated in red text below the X-axis. One RM (37072) became infected after 1 low dose (10^4^ PFU) inoculation, one RM (37812) became infected after 1 intermediate dose (10^5^ PFU) inoculation, one RM (37812) became infected after 2 high dose (10^6^ PFU) inoculations and another RM (40125) became infected after 5 high dose (10^6^ PFU) inoculations. Plasma vRNA was detectable by day 4–6 PI and was cleared in 7–10 days.

We produced a high titer ZIKV stock from the plasma of a Brazilian blood donor [28] by short-term culture on Vero cells (ATCC, Manassas,VA). The plasma was an aliquot of the same plasma sample from which strain Zika virus/H.sapiens-tc/BRA/2015/Brazil_SPH2015 was isolated [28]. The ZIKV stock contained approximately 10^7^ PFU/ml of infectious virus when titrated by Vero cell plaque assay and approximately 6x10^9^ vRNA copies/ml by the Taqman RT-PCR described below. The atraumatic virus inoculation procedure consisted of inserting a 1 CC needless tuberculin syringe containing 1 ml of the ZIKV stock into the vagina until the tip touched the cervix. Then the syringe was gently withdrawn while the viral inoculum was expelled. This procedure was repeated weekly until an animal was plasma ZIKV RNA+ on 2 consecutive time points (Fig 1). Animals that remained uninfected after 8 vaginal ZIKV inoculations were treated with Depoprovera using published protocols [29] that have been used to enhance vaginal SIV transmission in RM. Briefly, 4 weeks before, and on the day of, challenge with ZIKV, 30 mg of Depo-Provera [29] was administered by intramuscular injection.

### Nucleotide sequencing of the ZIKV stock

The Zika virus inoculum was sequenced in duplicate using a method adapted from Quick et. al. [30]. Briefly, viral RNA was isolated from 1 ml of cell culture supernatant using the Maxwell 16 Total Viral Nucleic Acid Purification kit. Approximately 1.4x10^5^ viral RNA templates were converted into cDNA using the SuperScript IV Reverse Transcriptase enzyme. The cDNA was then split into two multi-plex PCR reactions using the PCR primers described in Quick et. al with the Q5 High-Fidelity DNA Polymerase enzyme. PCR products were then tagged with the Illumina TruSeq Nano HT kit and sequenced with a 2 x 300 kit on an Illumina MiSeq. Fastq reads were analyzed using a series of custom scripts generated in Python, as follows. First, up to 1000 reads spanning each of 35 amplicons were extracted from the data set. Extracted reads were then mapped to the Zika reference for PRVABC59 and Zika virus (strain Zika virus/H.sapiens-tc/BRA/2015/Brazil_SPH2015). Variant nucleotides were then called using SNPeff, using a 5% cutoff. The output.vcf and.bam files could be interrogated in Geneious and differences between the inocula and reference strains could be determined.

### Blood, urine and cervicovaginal lavage (CVL) sample collection and RNA isolation

Blood was collected from the femoral vein by venipuncture 3–4 times a week, on the day of ZIKV inoculation and, 2, 4, and often 6, days later. Urine samples were collected from pans placed under the animals’ cages on the days that blood samples were collected. Cervicovaginal lavages (CVL) were also collected on the days blood samples were collected by vigorously infusing 1–2 ml of sterile PBS into the vaginal canal and aspirating as much of the instilled volume as possible. Care was taken to insure that the cervical mucus was included in the lavage fluid and that no trauma to the mucosa occurred during the procedure. One half of the CVL sample was snap frozen on dry ice and stored at −80°C until analysis. The remainder was spun and the resulting cell pellet was used RNA isolation. The supernatant was treated with 10× Protease Inhibitor (Roche/Sigma Aldrich, St Louis Mo) and subsequently used for cytokine and chemokine quantitation. The lavage for sample collection and the preparation procedure resulted in at least a 10-fold dilution of the cervicovaginal secretions. RNA was isolated from 1 ml urine, EDTA blood plasma, or CVL by QIAamp UltraSens Virus Kit (Qiagen, Redwood City CA) following the manufacturer’s protocol.

### Tissue collection and sample preparation

Genital tract tissues (vulva, vagina, cervix, uterus, ovary) and genital lymph nodes (inguinal, obturator and iliac lymph nodes), gut tissues (duodenum, jejunum, ileum, colon and mesenteric lymph nodes), oral tissues (lip/cheek pouch, tonsil, tongue, parotid salivary gland) distal lymphoid tissues (axillary, bronchial lymph nodes and spleen), urinary tract (bladder, kidney), CNS (Frontal cortex, temporal lobe, eye), cerebrospinal fluid (CSF) and blood were collected at the time of necropsy and analyzed for ZIKV RNA levels. Tissues were stored in RNAlater (Ambion, Austin, TX) and kept at -20°C until preparation of RNA. Tissues stored at -20°C thawed and removed from RNAlater were diced with a razor blade in a sterile petri dish as small as possible. Tissues fragments were then placed into 2.0ml screw cap Sarstedt tubes with 1 x 7mm stainless beads (Qiagen, Redwood City CA) added per tube with 600ul RLT Buffer and shaken 5 mins in bead beater to homogenize. The homogenates were processed using the Qiagen RNeasy Mini Kit (Qiagen, Redwood City CA) to extract total RNA with optional DNase treatment on column per manufacturer’s instructions. Skin and fibrous tissues were treated with additional proteinase K digestion as described in Appendix C of the kit handbook. Brain samples required 5ul of Reagent DX to prevent excessive foaming.

### Zika virus isolation

We used monolayers of Vero cells (ATCC, Manassas, VA) to isolate infectious virus from selected tissue samples collected from the ZIKV-inoculated animals at necropsy. Briefly, up to 10^7^,tissue mononuclear cells isolated from tissues were added to a confluent monolayer of Vero cells in 6-well plates (Costar Inc., Cambridge, MA) for tissues yielding <10^6^ cells), or T25 flasks (Costar Inc.) for tissues yielding > 10^6^ cells. The co-cultures were incubated at 37°C and culture supernatants were harvested at 2, 4 and 7 days after initiation. The supernatants were assayed for the presence of ZIKV RNA by qRT-PCR (described below). A sample was considered to be positive for infectious virus if the vRNA levels steadily increased in supernatants of the corresponding co-culture. No effort was made to titer the levels of infectious virus in samples.

### vRNA quantitation by quantitative real time polymerase chain reaction (qRT-PCR)

For urine, plasma, and CVL samples, 25ul of eluted RNA was converted to cDNA with Superscript III (Thermo Fisher Scientific, Waltham, MA) using random primers in a 60ul reaction and quantified in quadruplicate by qPCR on an Applied Biosystems QuantStudio 6 Flex Real-Time PCR System using 2x Universal Taqman Master Mix (Thermo Fisher Scientific, Waltham, MA) with published primers and probe that target the ZIKV E glycoprotein from Lanciotti et al [17] (forward 5’-CGYTGCCCAACACAAGG-3’, reverse 5’-CACYAAYGTTCTTTTGCABACAT-3’, and probe 5’-6fam AGCCTACCTTGAYAAGCARTCAGACACYCAA-BHQ1-3’). All RNA samples were tested in 4 replicate PCR reactions carried out in 96-well optical plates (Applied Biosystems, Foster City, CA). All PCR reactions included primers and probes for GAPDH to detect problems with the assay or RNA isolation and all plates contained several wells that held only 25μl nuclease free water to detect contamination. Standard curves for the ZIKV E glycoprotein primers and probe assay were generated on every plate by making 10-fold dilutions of a purified 444bp E glycoprotein PCR fragment starting at a known concentration. The 444bp PCR fragment was generated for this purpose by PCR amplification from ZIKV stock cDNA using primers z_F: 5’-CATACAGCATCAGGTGCATAGGAG-3’, z_R: 5’-AGCCATGAACTGACAGCATTATCC-3’ with Phusion HotStart II DNA Polymerase (Thermo Fisher Scientific, Waltham, MA). The fragment was purified with QIAquick PCR Purification Kit (Qiagen, Redwood City CA) and the concentration calculated using the average of 6 independent spectrophotometer readings (Nanodrop/\, Thermo Fisher Scientific, Waltham, MA). Five 96-well plates of individually serially diluted standard curves with concentrations ranging from 10^7^ copies/well to 1 copy/well were run to generate the line equation used to analyze all qPCR assays. For each 96 well plate, 11 wells of each dilution were run including positive and no-template controls. When the dilution of this fragment is done correctly, we generate 7–10 positive wells out of 10 at the 10-copy range and 2–3 positive wells out of 10 in the single copy range. Thus, the assay can detect a single copy of ZIKV env cDNA per well. To determine the sensitivity of the assay in actual samples, 10-fold serial dilutions of vRNA from the ZIKV stock were added to plasma or RNA extracted from a mesenteric LN collected from a ZIKV negative RM. The assay was negative when 10–15 copies of ZIKV RNA were added to the cDNA synthesis reaction, which results in about 1 vRNA copy in each well. However, 6 of 6 wells were positive when 100–150 copies of ZIKV RNA were added to the cDNA synthesis reaction, which is equivalent to 10–13 copies of vRNA in each well. There was no amplification of ZIKV E glycoprotein sequences from the RNA isolated from any plasma or tissue samples from ZIKV negative animals. Thus, we estimate that the limit of quantitation in this ZIKV E glycoprotein PCR assay is 120 vRNA copies/ml of CVL, plasma or urine. While in tissue samples, the limit of quantitation is 33 vRNA copies/ug of total tissue RNA analyzed. Viral load data from plasma, urine, and CVL are expressed as vRNA copies/ml. Viral load data from tissues are expressed as vRNA copies/ug total RNA.

### ZIKV-specific antibodies detected by ELISA

A commercial ELISA kit was used to test for the presence of ZIKV-specific-antibodies in plasma and CVL of inoculated animals. The NHP Zika virus serology test kit (XpressBio, Frederick MD) uses a Ugandan ZIKV NS1 protein as the capture antigen. There is about 97.5% amino acid identity between the Ugandan virus and contemporary circulating Asian ZIKV virus strains in the NS1 region. The kit was used as directed by the manufacture to test plasma samples. CVL samples, processed as described above, were diluted 1:1 and 1:2 and tested with the same kit.

### Measurement of cytokine and chemokine levels in plasma and CVL

Twenty-nine cytokines, chemokines and growth factors were measured in plasma and CVL samples using the Monkey Cytokine Magnetic 29-Plex Panel for the Luminex (Invitrogen, Carlsbad CA) according to the manufacturer's instructions. The analytes measured included IL-1β, IL-1RA, IL-2, IL-6, IFN-γ, IL-12, CCL3, CCL5, CCL11, CXCL8, CXCL9, CXCL10, CXCL11, and MIF. EDTA-plasma samples were diluted up to four fold with assay diluent and CVL samples were diluted up to 4 fold with a 1:1 mixture of PBS and assay diluent. Samples were incubated with antibody-coupled beads for 2 hours at room temperature, followed by incubation with a biotinylated detection antibody for 1 hour and streptavidin-phycoerythrin for 30 minutes. Each sample was assayed in duplicate, and cytokine standards supplied by the manufacturer were run on each plate. Multianalyte profiling was performed using a Luminex-100 system, and data were analyzed using Miliplex analyst software, version 5.1 (Millipore/Fisher Scientific, Waltham, MA). The median level of each analyte in a sample is reported. For these analytes, the sensitivity of the assay ranges from 0.5–20 pg/ml plasma according to the manufacturer.

### Data analysis

GraphPad Prism version 5 for Apple OSX10.4 (GraphPad Software, San Diego California USA) and Macintosh computers (Apple Inc., Cupertino CA) were used for statistical analysis and graphing the data.

### Accession numbers

Zika virus strain PRVABC59; genbank accession number KU501215

Zika virus strain/H.sapiens-tc/BRA/2015/Brazil_SPH2015; genbank accession number KU321639.1

## Results

### Vaginal transmission of ZIKV

We produced a high titer (10^7^ PFU/ml/6x10^9^ vRNA copies/ml) ZIKV stock by culturing the plasma of a Brazilian blood donor [28] on Vero cells. The isolate was confirmed by next generation sequencing to be an Asian-lineage ZIKV. We mapped the sequences to the Zika-PRVABC59; genbank accession number KU501215 and Zika virus/H.sapiens-tc/BRA/2015/Brazil_SPH2015; genbank accession number KU321639.1. We found that nucleotide and AA sequence of the major variant in our Zika virus stock was identical to the Brazilian KU321639.1 reference sequence but had 35 positions with fixed nucleotide differences compared to the Puerto Rican KU501215 reference sequence. There were two other minor variants present in the stock at a frequency of between 5–10%; the defining nucleotide differences were not in a location of repeated nucleotides. Thus, our ZIKV stock is essentially clonal as it contains only a few infrequent variations from a single ZIKV sequence.

**Fig 2:**
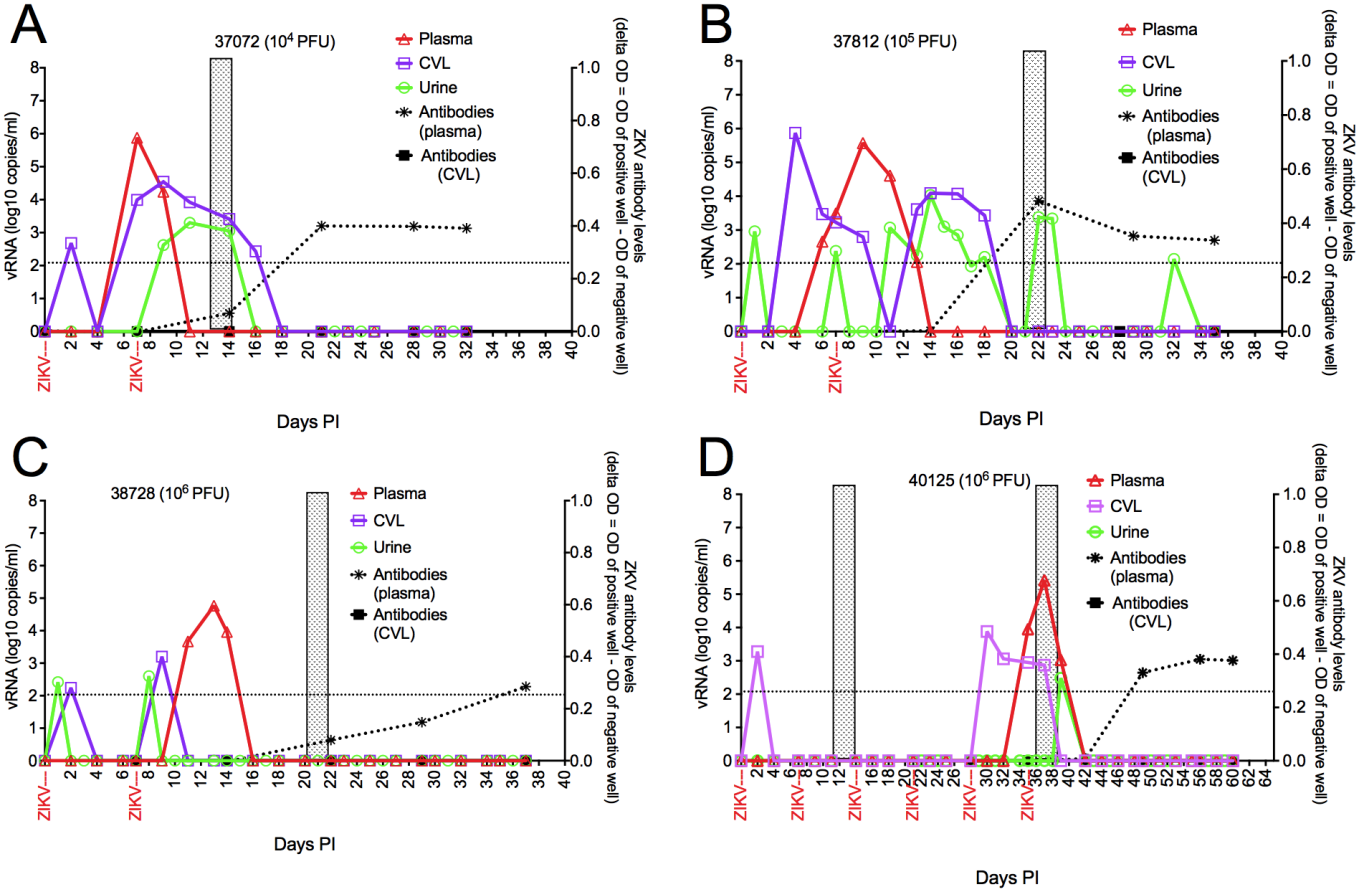
Detailed virology and serology of 4 RM infected after vaginal ZIKV inoculation in relation to the menstrual cycles of each animal. vRNA levels in plasma, CVL, urine and packed blood cells (whole blood) on the left y-axis. Plasma anti-ZIKV antibody levels are indicated on the right y-axis. The shaded vertical boxes indicate the days menstrual blood was detected by visual inspection, with the first day of bleeding designated Day 1 of the menstrual cycle. A) RM 37072 infected after 1 low dose (10^4^ pfu) inoculation in the peri-ovulatory phase of her menstrual cycle. B) RM 37182 infected after 1 moderate dose (10^5^ pfu) inoculation in the follicular phase of her menstrual cycle. C) RM 38728 infected after the second of 2 high dose (10^6^ pfu) inoculations in the peri-ovulatory phase of her menstrual cycle. D) RM 40125 infected after 5^th^ of 5 high dose (10^6^ pfu) inoculations in the luteal phase of her menstrual cycle.

**Fig 3:**
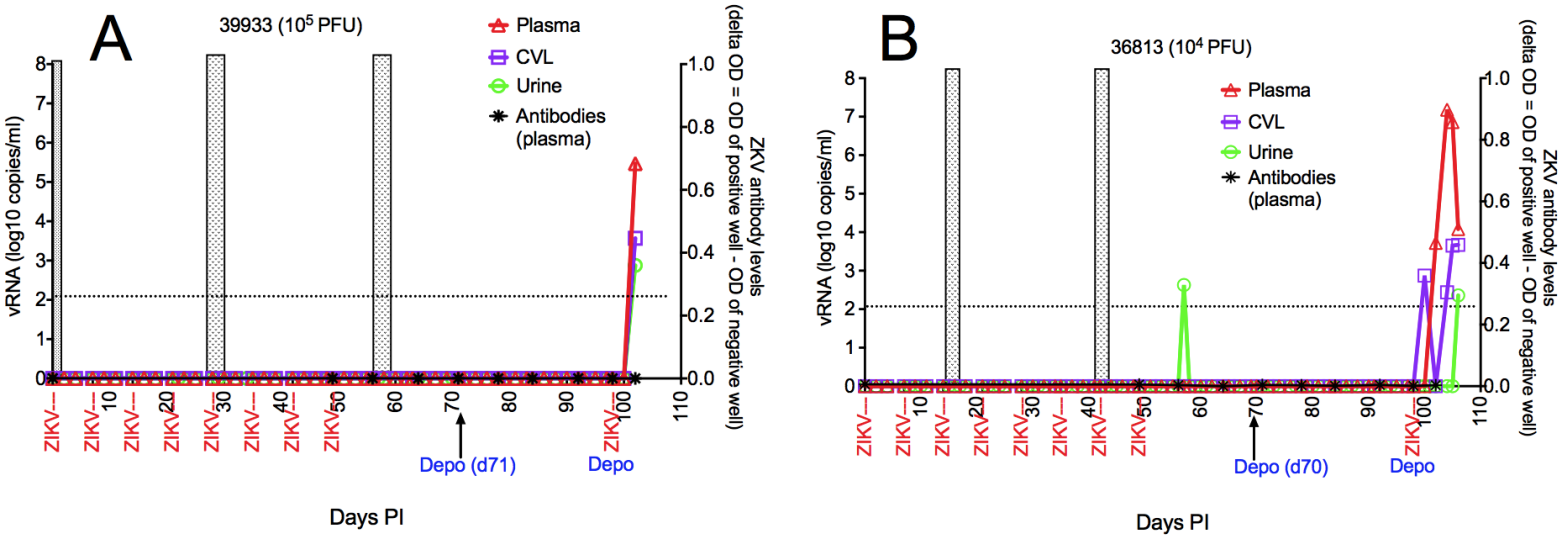
Detailed virology of 2 Depoprovera-treated RM infected after vaginal ZIKV inoculation. vRNA levels in plasma, CVL and urine on the left y-axis. Plasma anti-ZIKV antibody levels are indicated on the right y-axis. The shaded vertical boxes indicate the days menstrual blood was detected by visual inspection, with the first day of bleeding designated Day 1 of the menstrual cycle. After Depoprovera treatment, A) RM 39933 became infected after 1 moderate dose (10^5^ pfu) inoculation and B) RM 36813 became infected after 1 low dose (10^4^ pfu) inoculation. The timing of the ZIKV inoculations and Depoprovera injections is indicated under the x-axis.

It has been reported that the dose of WNV or Dengue virus in an infected mosquito bite ranges from 10^4^−10^6^ PFU [31,32]. While the level of infectious ZIKV in semen is unknown, ZIKV RNA levels of 10 ^7^–10 ^8^ vRNA copies/ ml semen have been reported [4,33]. As one of the purposes of our study was to define the dose of ZIKV required for vaginal transmission, we chose to use a similar range of ZIKV doses for vaginal inoculation of RM. Thus, two animals were vaginally inoculated weekly with10^4^ PFU (6x10^6^ vRNA copies), two animals were inoculated with 10^5^ PFU (6 x 10^7^ vRNA copies) and two animals were inoculated with 10^6^ PFU (6 x 10^8^ vRNA copies). There was a 7-day interval between each vaginal inoculation (Fig 1). Two RM became infected (plasma ZIKV RNA+) after 1 vaginal inoculation with ZIKV (Fig 1). One (37812) of these 2 RM was exposed to a moderate virus dose (10^5^ PFU/6 x 10^7^ vRNA copies) in the luteal phase of the cycle (approx. cycle day 21) (Fig 2B) and the other (37072) to a low dose (10^4^ PFU/6 x 10^6^ vRNA copies) of ZIKV in the peri-ovulatory phase of the cycle (approx. cycle day 15) (Fig 2A). A third RM (37828) became infected after 2 high dose (10^6^ PFU/6x10^8^ vRNA copies) vaginal ZIKV inoculations with transmission occurring after the 2^nd^ inoculation in peri-ovulatory phase of the cycle (approx. cycle day 14) (Fig 2C), and another RM (40125) after 5 vaginal inoculations with a high dose (10^6^ PFU/6 x 10^8^ vRNA copies) of ZIKV with transmission occurring after the 2^nd^ inoculation in follicular phase of the cycle (approx. cycle day 7) (Fig 2D). Finally, after 8 weekly vaginal ZIKV inoculations, one low dose RM (36813) and one moderate dose RM (39933) remained uninfected. Both of these animals were treated with Depoprovera and 30 days later they were re-inoculated vaginally with the same dose of ZIKV they were previously inoculated with 8 times without transmission. After Depoprovera treatment, both of these RM became infected after 1 vaginal ZIKV inoculation (Figs 1 and 3). Thus, RM were readily infected with ZIKV after vaginal inoculation with a concentration of ZIKV within the range that is found in human semen [4,33].

### Replication kinetics and dissemination of ZIKV after vaginal transmission

In all 4 Depoprovera-naive RM, plasma ZIKV RNA was first detected at 4 or 6 days post-inoculation (PI), reached peak levels at 6–10 days PI and was undetectable by 9–14 days PI. The mean duration of virema was 8.2 days (Fig 2A–2D). ZIKV RNA levels in CVL and urine were also determined.

In 3 of 4 Depoprovera-naive RM, a blip of vRNA was detected in CVL 24–48 hours after vaginal inoculation, and then vRNA became undetectable (Fig 2A, 2C and 2D). In 2 of these RM, vRNA reappeared in CVL before plasma vRNA was detectable (Fig 2C and 2D). In the fourth RM, high and sustained levels of ZIKV RNA were found in CVL beginning at 3 days PI, prior to detection of plasma vRNA (Fig 2B). Among all 4 Depoprovera-naive RM, CVL ZIKV RNA was detected at 2–6 days PI, peaked at 2–9 days PI and was undetectable by 12–21 days PI. The mean duration of ZIKV RNA shedding in CVL was 8.1 days (Fig 2A–2D).

In 2 of 4 RM, a blip of vRNA was detected in urine within 24–48 hours post-inoculation (PI) (Fig 2B and 2C), with vRNA reappearing in urine before plasma vRNA was detectable in 1 of these 2 RM (Fig 2C). In the other 2 RM, high and sustained levels of ZIKV RNA were found in urine beginning at 9–12 days PI, long after detection of plasma vRNA. Among all 4 RM, urine ZIKV RNA was detected by 1–11 days PI, peak levels occurred at 7–14 days PI and vRNA was undetectable in urine by 9–32 days PI, (mean duration of urine ZIKV RNA shedding: 6 days) (Fig 2A–2D).

The detailed virology of the 2 RM that resisted systemic infection, despite 8 vaginal ZIKV inoculations spanning 2 menstrual cycles, until they were treated with Depoprovera is shown in Fig 3. On day 57 PI, 8 days after the last ZIKV vaginal inoculation on Day 49 PI and before DepoProvera treatment, vRNA was detected in one urine sample, but not plasma or CVL, of RM 36813 (Fig 3B). However following Depoprovera treatment, vRNA was present in CVL 2 days after the vaginal ZIKV rechallenge on day 100, while plasma vRNA was detected 2 days later on day 102 (Fig 3B). In the other Depo-treated RM, (39933) ZIKV RNA was first detected in plasma, CVL and urine on day 102, 4 days after vaginal ZIKV inoculation (Fig 3A).

### Innate antiviral and pro-inflammatory responses in plasma and FRT after vaginal ZIKV transmission

**Fig 4:**
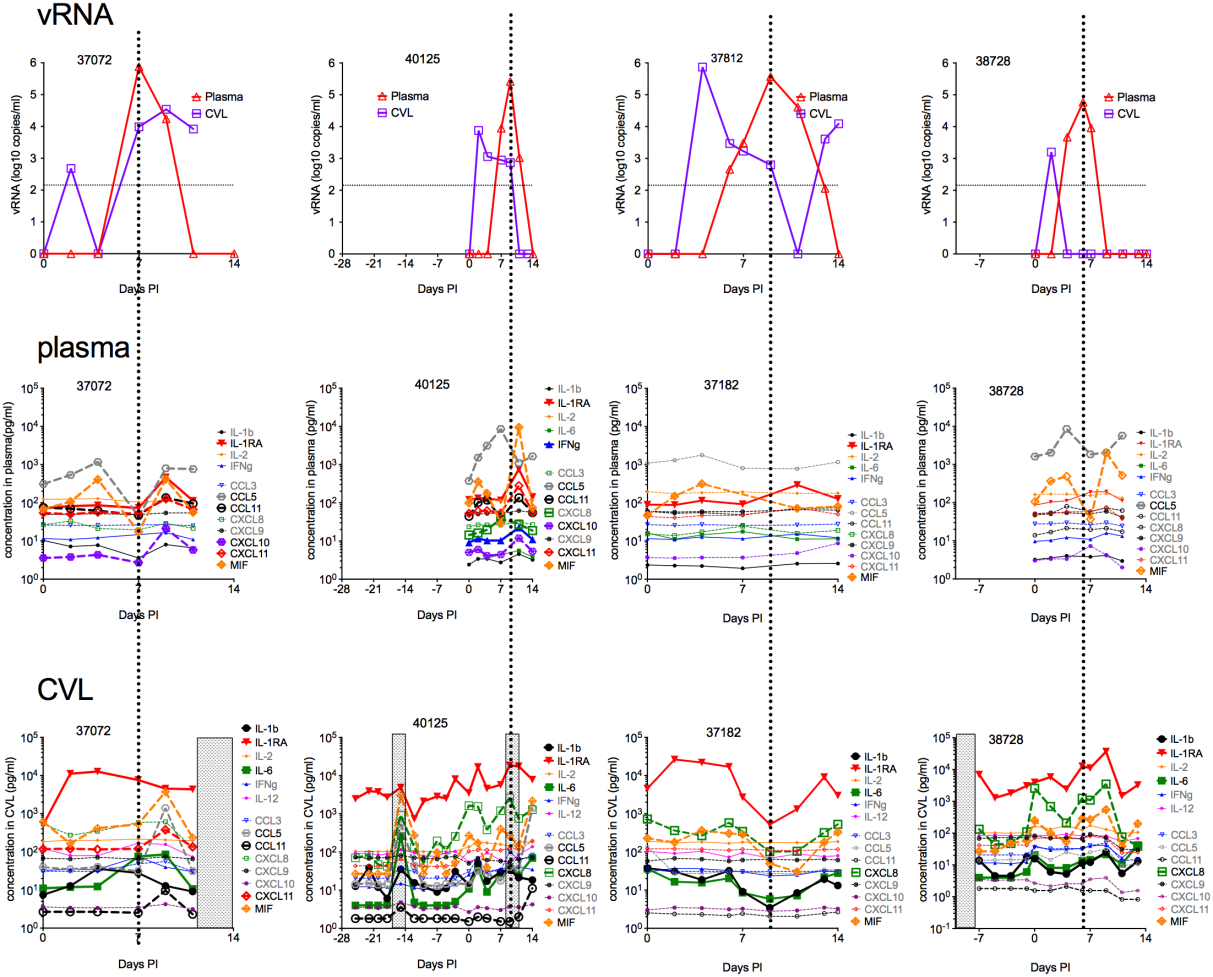
Cytokine and chemokine levels in plasma and CVL from 4 ZIKV-infected female RM. The top row is plots of the vRNA levels in plasma and CVL samples for each RM. The middle row is plots of the cytokine and CC levels in plasma samples. The bottom row is plots of the cytokine and CC levels in paired CVL samples. Each column is data from one animal. The x-axis on every graph indicates the day of sampling relative to the day of transmission designated Day 0. The vertical dashed lines indicate the timing of peak plasma vRNA levels. The shaded vertical boxes indicate the days menstrual blood was detected by visual inspection, with the first day of bleeding designated Day 1 of the menstrual cycle. The concentrations of the analytes in bold face and with larger symbols varied from baseline by about 10-fold after infection.

We used a Luminex-based bead array assay to assess changes in the levels on cytokines and chemokines in the plasma and CVL in the 4 DepoProvera naive RM (Fig 4). All 4 RM (37072,40125,37182,38728) had clear increases in the level of macrophage inhibitory factor (MIF) in plasma. In addition 3 of 4 RM had increased plasma levels of L-1RA (37072,40125,37182), and CCL5 (RANTES) (37072,40125,38728), and 2 of 4 RM (37072,40125) had increased plasma levels of CCL11 (Eotaxin), CXCL10 (IP-10) and CXC11 (I-TAC) (Fig 4). The plasma levels of these mediators both increased and decreased after infection, but the highest levels of an analyte were generally found in plasma samples collected the day after peak vRNA levels and the lowest levels of most analytes were found in plasma samples with low vRNA levels (Fig 4). The pattern of changes in MIF levels were unique in that they increased prior to, or just after, initial detection of plasma vRNA; were lowest at the peak in plasma vRNA levels; and, in 3 of 4 RM (37072,40125,38728), increased to their highest level days after the peak in plasma vRNA (Fig 4).

The effect of vaginal ZIKV transmission on cytokine and chemokine levels in CVL was more dramatic and was detectable prior to changes in plasma levels of these analytes (Fig 4). All 4 RM had clear changes in the levels of IL-1b, IL-1RA, IL-6 and macrophage inhibitory factor (MIF) in CVL (Fig 4). In addition, 3 of 4 RM (40125,37182,38728) had increased levels of CXCL8 (IL-8), and 2 of 4 RM (37072,40125) had increased levels of CCL5 (RANTES) and CCL11. The levels of these mediators in CVL both increased and decreased after infection, but the highest levels of most analytes were generally found in CVL samples with high vRNA levels and the lowest levels of most analytes were found in CVL samples with low vRNA levels (Fig 4). In 3 animals (37072,37182,38728), IL-1Ra levels increased on the first day vRNA was detected in CVL and remained elevated until vRNA levels dropped (Fig 4). Of note, the levels of IL-1b and IL-1Ra were 10–100 fold higher in CVL than plasma (Fig 4), despite the dilution that occurs when CVL samples are collected. CVL sample collection began on Day 0, just prior to the first ZIKV inoculation, and thus for the animals (37072,38728) that became infected after 1 ZIKV inoculation there was a single pre-infection sample, for the animal (38728) infected after 2 inoculations there were 1 week of pre-infection samples and for the animal (40125) infected after 5 inoculations 4 weeks of pre-infection samples are available. The cytokine levels in the pre-infection CVL of the latter 2 animals were relatively stable, except in the CVL samples collected during menses (day -18 to -14) from 40125, in which many of the analytes were elevated (Fig 4).

### ZIKV-specific antibodies were detected in plasma but not CVL after ZIKV infection

We used a commercial ELISA assay to assess the levels of ZIKV-specific antibodies in plasma and CVL of the ZIKV inoculated animals. For all 6 RM infected with ZIKV after vaginal inoculation (Fig 1), paired plasma and CVL samples collected weekly from the day of first ZIKV inoculation to necropsy were tested. Of the 4 RM that became infected without DepoProvera treatment, ZIKV-specific antibodies were detected in plasma of one (37072) 7 days after vaginal ZIKV transmission and 14 days after vaginal ZIKV transmission in the other 3 RM (37812, 38728, 40125) (Fig 2). ZIKV-specific antibodies were never detected in the CVL samples of any of these 4 animals (Fig 2s). The 2 RM that remained ZIKV negative after 8 vaginal inoculations but then became infected after DepoProvera treatment and 1 additional vaginal inoculation, remained anti-ZIKV plasma antibody negative from the day of the first ZIKV inoculation until necropsy in the acute stage of infection (Fig 3).

### ZIKV tropism and routes of dissemination after vaginal inoculation

**Fig 5:**
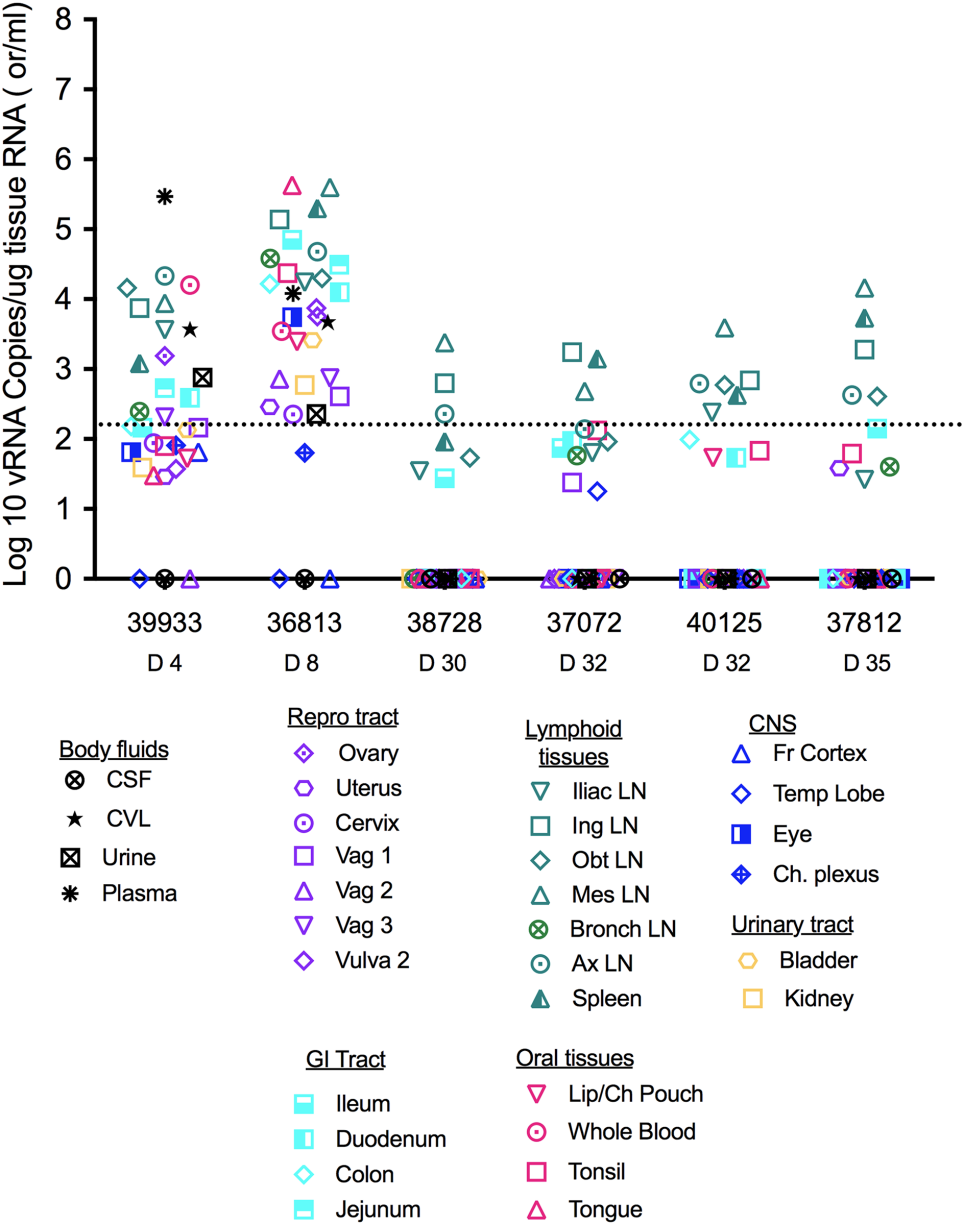
ZIKV RNA levels in tissues from 6 female RM infected with ZIKV by vaginal inoculation. Individual RM numbers are listed on the x-axis with the day of necropsy relative to the day of transmission. Each symbol is the result from a single tissues sample and the color and symbol shapes identify the tissues type and specific tissue, respectively. Note that both the RM necropsied in the acute stage were treated with Depoprovera prior to vaginal ZIKV inoculation.

To better understand the tissue tropism of ZIKV, we determined vRNA levels in tissues of all 6 RM infected with ZIKV by vaginal inoculation (Fig 5). The 2 RM treated with Depoprovera prior to infection (Fig 1) were necropsied at 4 and 8 days after vaginal ZIKV inoculation, when vRNA was detectable in plasma and CVL. At 4 days PI (39933), ZIKV RNA was present at low to moderate levels in the urinary tract, FRT and draining lymph nodes. vRNA was also detected in distal lymph nodes and spleen (Fig 5). At 8 days PI (36813), vRNA levels were 100–1000 fold higher in all tissues, with the highest levels in salivary glands and lymphoid tissues. ZIKV RNA was also detected in the central nervous system (CNS) at this early stage of infection (Fig 5). In addition, infectious ZIKV was isolated from 1 of 6 vRNA+ lymphoid tissues at 4 days PI (39933); while at 8 days PI (36813) ZIKV was isolated from 6 of 6 lymphoid tissues tested (Table 1).

**Table 1:** Results of ZIKV isolation assay in lymphoid tissues collected at necropsy.

Animal number	Days PI^a^	Tissue					
		Ax Ln	Ing Ln	Spleen	Obt. LN	Iliac LN	Mes LN
39933	4	**-**	**-**	**-**	**-**	**-**	**+**
36813	8	**+**	**+**	**+**	**+**	**+**	**+**
38728	30	**-**	**-**	**-**	**-**	**-**	**-**
37072	32	**-**	**-**	**-**	**-**	**-**	**-**
40125	32	**-**	**-**	**-**	**-**	**-**	**-**
37812	35	**-**	**-**	**-**	**-**	**-**	**-**

^a^ = number of days between ZIKV infection and tissue collection at necropsy.

The remaining 4 RM were necropsied between 30 and 35 days PI, about 2 weeks after vRNA was last detectable in plasma. At this stage, the RM were plasma ZIKV RNA negative and anti-ZIKV IgG positive. However, ZIKV RNA was detected at low to moderate levels in all lymphoid tissues tested from all 4 RM. In addition, low level ZIKV RNA was detected in the CNS (temporal lobe of brain) of one RM and the FRT (uterus) of a second RM (Fig 5). Zika RNA is also detected in tissues, including the brain and male and female reproductive tissues, during early and late stages of infection after SQ ZIKV inoculation of RM [34–36]. However, we were not able to recover infectious ZIKV from tissues of any of these 4 animals (Table 1). Thus, the significance of the ZIKV RNA that persists in tissues of RM long after it is cleared from plasma is unclear.

## Discussion

Given the severe disease ZIKV can cause in a developing fetus [37], the risk of transmission to women during pregnancy is of particular concern. Despite documented cases of ZIKV sexual transmission [1–13], the frequency and efficiency of sexual ZIKV transmission is unclear. Two modeling studies of ZIKV transmission dynamics in the recent outbreak in the Americas estimated that sexual transmission contributed between 3–45% to the overall basic reproduction number (*R*_0_) of ZIKV in a population [38] [39]. Obviously, this very wide range indicates that there is still considerable uncertainty about the significance of sexual transmission ZIKV in propagating and maintaining the virus in human populations [38] [39]. To better understand the potential for sexual transmission of ZIKV, a NHP model of vaginal transmission is needed. Macaques were experimentally infected with mouse-brain passaged ZIKV in the 1950s, however, until recently there were no published reports describing the biology of ZIKV infection in nonhuman primates. Since early 2016, animal models of human ZIKV have been developed using Type-1 IFN-antibody treated mice, Type-1 IFNR knockout mice [40–46] and RM [35,36,47–49]. To date, the reported non-human primate (NHP) studies have used intravenous (IV) or SQ routes of ZIKV inoculation to infect RM [35,36,47,48]. The data reported here demonstrate that ZIKV can be readily transmitted to mature cycling female RM by vaginal inoculation.

Perhaps, the most striking finding in this study is that the kinetics of virus replication and dissemination in RM after intravaginal ZIKV inoculation are markedly different than after SQ virus inoculation [34,36,49]. After SQ inoculation of RM with Asian lineage ZIKV, vRNA is detected in blood plasma as early as 1 d after infection and subsequently in both the urine and saliva [36,49]. The appearance of vRNA in urine and saliva is delayed and blunted when compared to plasma and ZIKV RNA was detected only infrequently in CVL of RM after SQ inoculation [36,49]. As in SQ inoculated RM, ZIKV shedding from the FRT is rare in ZIKV-infected women [50] the majority of whom were presumably infected by mosquito bite. In SQ inoculated RM, viral RNA is cleared from plasma and urine by day 10, but remains detectable in saliva and semen for more than 3 weeks [36]. In marked contrast, plasma vRNA is delayed by several days, and virus shedding from the FRT occurred, in all RM inoculated with ZIKV intravaginally (Fig 2). Of note, ZIKV is found in the FRT of a subset of infected women [51–53], and it is tempting to speculate that in these cases the virus was sexually acquired.

In addition to the delay in plasma vRNA in ZIKV vaginally inoculated RM compared to SQ infected RM, virus dissemination to tissues was slower and stepwise in the vaginally inoculated animals. Four days after vaginal inoculation, ZIKV RNA was present at low to moderate levels in the urinary tract, FRT, draining lymph nodes distal lymph nodes, spleen. However, at 8 days PI, vRNA levels were 100–1000 fold higher in all tissues, with the highest levels in salivary glands and lymphoid tissues indicating that the virus was still disseminating more than 1 week after infection. At 30 and 35 days PI, the vaginally infected RM were plasma ZIKV RNA negative but had low to moderate ZIKV RNA levels in all lymphoid tissues tested. In addition, low level ZIKV RNA was detected in the uterus of one of these 4 RM (Fig 5). Similarly, 7 days after SQ ZIKV inoculation high levels of ZIKV RNA were found in numerous tissues, including the brain and reproductive tract; and ZIKV RNA persisted through day 35 PI in neuronal, lymphoid and joint/muscle tissues [34,36]. However, while infectious ZIKV was isolated from multiple tissues at day 7 PI, infectious virus was not found in tissues collected at 28 days PI [34]. Thus, although ZIKV RNA seems to persist in target tissues for a considerable period after it is cleared from the blood, it remains to be seen if this persistent RNA contributes to pathogenesis or can serve as a reservoir for infectious virus.

In the RM infected by vaginal ZIKV inoculation, the levels ZIKV RNA in CVL was similar to plasma vRNA levels. Given the 10–100 fold dilution of cervicovaginal secretions that occurs during the CVL collection process, vRNA levels in CVL were at least equal to, and often higher than, plasma vRNA levels (Fig 2). Thus the FRT is able to support a high level of ZIKV replication. The timing of ZIKV shedding in CVL also demonstrated that virus replication in the FRT was independent of systemic replication. Often ZIKV RNA was detected in CVL before it appeared in plasma and ZIKV RNA could also be found in CVL after virus had been cleared from plasma. This suggests that the virus being shed in CVL is from local replication in the FRT that is independent of virus replication in other tissues. The presence of ZIKV in the FRT after its disappearance from blood and urine samples has also been documented in women [51,52], which suggests that the ZIKV preferentially replicates in the FRT of RM and women that acquire the infection through sex or vaginal inoculation.

There was substantial variability between the individual RM in susceptibility to infection after vaginal ZIKV inoculation in this study. It has been reported that the stage of the menstrual cycle at vaginal inoculation effects susceptibility to infection with SHIV and SIV in RM [54,55]Sodora. In these reports, susceptibility to viral infection was highest in menses and the luteal phase of the cycle [56]. In the current study, of the 4 ZIKV+ animals infected without Depo-Provera treatment, 37072 was infected in peri-ovulatory phase of the cycle (approx. cycle day 15); 38728 was infected in peri-ovulatory phase of the cycle (approx. cycle day 14); 37812 was infected in early luteal phase of the cycle (approx. cycle day 21); and 40125 was infected in follicular phase of the cycle (approx. cycle day 7) (Fig 2). Thus, there is no evidence that the stage of the menstrual cycle at exposure explains the variability vaginal ZIKV transmission in these 4 monkeys, however this initial observation needs to be confirmed in larger studies.

Depo-Provera, a brand of the injectable hormonal contraceptive depot-medroxyprogesterone acetate (DMPA), is the most widely used injectable contraceptive in the world. We chose to test the effects of Depoprovera on vaginal ZIKV transmission because DMPA treatment enhances infectivity of viruses in various rodent and nonhuman primate models of female genital tract infection [57–61]. In fact, progesterone treatment is needed to infect mice with ZIKV by vaginal inoculation [46]. In addition, some observational studies identified DMPA as a significant risk factor for acquisition of HIV and other sexually transmitted infections (STI) in women, while other studies failed to detect this association [62–65]. Our observation that, after Depoprovera treatment, both of the RM that initially resisted vaginal ZIKV transmission became infected with one vaginal ZIKV inoculation is consistent with the conclusion that Depoprovera enhanced susceptibility to vaginal ZIKV transmission. Caution is warranted in interpreting our study however as only 2 animals were treated with Depo-Provera in the study.

Several mechanisms have been proposed to explain enhanced STI acquisition with Depo-Provera including mucosal epithelium thinning, enhanced tissue inflammation, suppressed cell-mediated immune responses, and altered vaginal microbiota. However, none of these putative biological mechanisms are experimentally proven [66,67]. It was recently reported that Depoprovera use in women is associated with increased hemoglobin, immune activation markers (HBD, HBB, IL36G), and decreased epithelial repair proteins (TFF3, F11R) in reproductive tract secretions [68]. Further, in mice Depo-Provera reduced expression of the desmosomal cadherin desmoglein-1α in the genital epithelium, enhanced inflammatory cells numbers in genital tissue by increasing mucosal epithelial permeability, and increased susceptibility to HSV-2 infection [69]. The results of both of these recent studies suggest that Depo-provera mediated increases in mucosal permeability facilitate endogenous vaginal microbiota invasion and tissue inflammation by breaking down the epithelial barrier. Thus the most likely explanation for enhanced vaginal ZIKV virus transmission in the Depo-Provera treated animals is that increased permeability of the vaginal mucosa allowed the virus inoculum to access more target cells in the lamina propria.

Although our ZIKV inoculum was delivered to monkeys as cell-free virions suspended in tissue culture fluid, women are exposed to ZIKV virions in semen, which may affect virus transmission. Seminal plasma (SP) has a basic pH that neutralizes the acidic pH of the vagina, thus seminal plasma may limit the inactivation of ZIKV deposited into the vagina. In fact, it has been shown that SP boosts SIV and HIV-1 infection in vitro and semen amyloid proteins contribute to this activity [70–74]. However, the significance of the in-vitro observations is unclear, as the addition of semen, SP or semen amyloid proteins does not dramatically enhance vaginal SIV transmission [75]. However, it has been reported that SP marginally increases vaginal SIV transmission if low-dose viral inoculums are used [76,77]. In addition, human and macaque seminal plasma are complex biologic fluids that vary substantially in chemical composition between individuals, and between individual ejaculates making it impossible to replicate experiments without using an aliquot of the semen sample used in the original experiment. Due to the limited volume, it is not possible to use the same human or macaque seminal plasma material for more than a few experimental vaginal inoculations. These technical factors make it impractical to use seminal plasma in animal experiments modeling vaginal virus transmission if reproducible results are desired. To insure the reproducibility of the results in the studies reported here, we did not include seminal plasma in the inoculum.

RM infected by SQ inoculation with ZIKV during the first trimester of pregnancy have persistent plasma vRNA, leading to the hypothesis that the fetus or placenta may be the source of persistent virus replication in the immune suppressed pregnant female [49]. This conclusion is consistent with a report that the placental/fetal tissues from 24 of 44 women suspected of being infected with Zika virus during pregnancy were positive for ZIKV RNA by RT-PCR. [78]. However the results reported here, and previous results in RM [34] and women [51,52], demonstrate that ZIKV RNA persists in the FRT and lymphoid tissues in non-pregnant RM and these tissues are another possible source of persistent plasma vRNA in pregnant animals.

Although we detected anti-ZIKV IgG antibodies in plasma of all 4 animals infected for more than 10 days with ZIKV. We did not detect anti-ZIKV IgG antibodies in CVL of any animals. This is unexpected as antiviral antibodies are routinely found in CVL of RM and women [79–82]. It is possible that this result is due to a technical issue with the commercial ELISA kit we used. We are in the process of developing ELISA assays to measure anti-ZIKV IgG subclass and IgA antibody responses and these assays will clarify whether anti ZIKV antibody responses that were undetectable by the commercial assay are present in the CVL. Our inability to detect a plasma antibody response in the 2 animals inoculated 8 times with ZIKV is consistent the lack plasma vRNA in the animals and confirms that they remained uninfected despite the repeated ZIKV exposures. Apparently, in the absence of infection, the amount of ZIKV antigen in the inoculum is insufficient to elicit a systemic antibody response when placed on the mucosal surfaces of the FRT.

The systemic cytokine response is minimal after SQ ZIKV inoculation of RM [34], and it was suggested that the low levels of cytokine activation in vivo may be the result ZIKV inhibiting the innate immune pathways that direct synthesis and secretion of pro-inflammatory cytokines [34]. However, we found evidence that vaginal ZIKV transmission and subsequent systemic infection results in an acute inflammatory response characterized by increases in pro-inflammatory cytokines and chemokines in CVL and, to a lesser degree, plasma. Further, after vaginal ZIKV transmission, the inflammatory response in the FRT corresponded temporally to periods of local ZIKV replication. Thus, peak levels of ZIKV shedding/replication in the FRT were often associated with increased levels of pro-inflammatory cytokines (IL-1b, IL-6), anti-inflammatory mediators (IL-1RA) and a subset of chemokines in CVL. These changes are consistent with an acute antiviral inflammatory response to local ZIKV replication and viral mediated tissue damage in the FRT. However, the pattern and levels of the inflammatory mediators were very different in the blood and CVL. MIF and IL1Ra were elevated in both plasma and CVL of 3 of 4 RM. While IL-6 were elevated in CVL, but not in plasma, (Fig 4) all animals and CXCL8 was elevated in CVL but not plasma of 3 of 4 animals. In addition, given the 10-fold dilution of secretions that occurs during the collection of CVL, the concentration of all these inflammatory mediators was generally higher in CVL than plasma. Thus, after vaginal ZIKV transmission, there was an obvious local and systemic inflammatory response that was delayed and enhanced compared to that reported in SQ inoculated RM [34]. This finding suggests that the pathogenesis of ZIKV disease can vary with the route of transmission. Taken together, the distinct timing and nature of the inflammatory response in the FRT compared to blood and the unique pattern of virology in the FRT, is consistent with the conclusion that ZIKV replication in the FRT is independent of replication in the systemic compartment.

The pattern of inflammation in the FRT and systemic compartments also provides considerable insight into ZIKV pathogenesis. MIF, the only cytokine that was elevated in both plasma and CVL samples of all 4 animals. DENV infection induces MIF production and secretion and secreted MIF can enhance DENV replication and increase vascular leakage through autophagy [83]. Thus MIF may contribute to inflammation and hemostatic abnormality during DENV infection [84] and there is a correlation between MIF serum levels and disease severity in dengue patients [85]. The high concentrations of IL-1b and CXCL8 in the CVL after ZIKV infection suggest that enhanced neutrophil recruitment is a major response to ZIKV replication in the FRT [86–88]. Recruitment of neutrophils i requires the upregulation and release of IL-1β [89,90] and IL-1 also markedly prolongs the lifespan and stimulates the effector function of neutrophils and macrophages [91]. IL1Ra was elevated in the plasma of 3 of 4 RM and the CVL of all RM. Of note, the levels of IL-1Ra were 10–100 fold higher in CVL than plasma (Fig 4). IL-1Ra competes with IL-1 for binding to the IL-1 receptor, blocking IL-1–induced pro-inflammatory signaling, and thus, may affect viral pathogenicity. Elevated levels of IL-1Ra have been described in humans with a number of viral infectious diseases [92–94], but the role of IL-1Ra in viral pathogenesis is unclear. Changes in the levels of IL-6 were found in the CVL of all 4 animals tested. Although, IL-6 is considered a marker of inflammation L-6 levels do not necessarily correlate with the levels of other inflammatory cytokines and IL-6 directly affects the adaptive antiviral immune response. IL-6 affects differentiation of CD4 T cells [95] and can also modulate aspects of the innate immune response to viral infection [96–98].

The findings that ZIKV shedding in CVL is not related to plasma vRNA levels and that a local inflammatory response develops in the FRT that is distinct from the systemic response is consistent with the conclusion that ZIKV replicates, and persists, in the FRT independent of the systemic ZIKV infection. This conclusion is also supported by observation that after vaginal ZIKV inoculation of IFNR^+/+^ mice, ZIKV replicates in the FRT but not in systemic tissues [43]. Thus, data from both the NHP and mice models of vaginal ZIKV transmission support the conclusion that, after vaginal ZIKV transmission the virus preferentially replicates in the FRT independent of replication levels in other tissues. The unusual tropism of ZIKV for the FRT raises the possibility of additional unexpected effects of vaginal ZIKV transmission, including the potential for enhanced fetal infection and pathology. In addition, it remains to be shown that a vaccine that protects animal models from mosquito transmitted ZIKV can protect against vaginal ZIKV transmission.
